# Cardiorespiratory Fitness, Functional Fitness and Body Composition Among Breast Cancer Survivors With 8 Weeks of Exercise Training: A Randomised, Controlled Non‐Inferiority Trial Comparing Remotely‐Supported and Partly‐Supervised Interventions

**DOI:** 10.1002/cam4.71608

**Published:** 2026-02-08

**Authors:** Lauren Struszczak, Jean‐Philippe Walhin, James Betts, Dylan Thompson, Mark Beresford, James E. Turner

**Affiliations:** ^1^ Centre for Nutrition, Exercise and Metabolism, Department for Health University of Bath Bath UK; ^2^ Public Health and Sports Sciences University of Exeter Exeter UK; ^3^ Royal United Hospitals Bath NHS Foundation Trust Combe Park Bath UK; ^4^ School of Sport, Exercise and Rehabilitation Sciences University of Birmingham Birmingham UK

**Keywords:** breast cancer survivors, exercise, fitness, health

## Abstract

**Background:**

This randomised, controlled non‐inferiority trial investigated whether 8 weeks of remotely‐supported exercise training changes cardiorespiratory fitness, functional fitness and body composition by a magnitude that is not meaningfully inferior to changes caused by partly‐supervised exercise training.

**Methods:**

Thirty female breast cancer survivors (57 ± 6 years, $\dot{V}O_{2}\max$ 28.9 ± 6.1 mL·kg^−1^·min^−1^, BMI 25.3 ± 3.3 kg·m^−2^) were randomised to 8 weeks of partly‐supervised (*n* = 15) or remotely‐supported (*n* = 15) exercise training. The partly‐supervised group undertook two supervised and one unsupervised session per week, progressing from 55% to 70% $\dot{V}O_{2}\max$ and 35–50 min. The remotely‐supported group were prescribed the same total duration of exercise per week (progressing from 105 to 150 min). Intensity was prescribed using heart rate targets corresponding to 55%–70% $\dot{V}O_{2}\max$. $\dot{V}O_{2}\max$, functional fitness, body composition and blood pressure were assessed pre‐ and post‐intervention.

**Results:**

Adherence was higher in the partly‐supervised group (87% ± 7%) versus the remotely‐supported group (64% ± 25%; *p* = 0.01). The remotely‐supported group exhibited changes in timed up and go (difference to partly‐supervised; 95% CI −0.8 to 0.4 s) and percentage body fat (difference to partly‐supervised; 95% CI −0.6 to 0.5 kg·m^−2^) that were non‐inferior to the partly‐supervised group. It was inconclusive whether changes among the remotely‐supported group for $\dot{V}O_{2}\max$ (difference to partly‐supervised; 95% CI −3.3 to 1.1 mL·kg^−1^·min^−1^), blood pressure (difference to partly‐supervised; 95% CI systolic; −3 to 12 mmHg, diastolic; −5 to 6 mmHg), 6 min walk (difference to partly‐supervised; 95% CI −54.0 to 0.4 m), or sit to stand (difference to partly‐supervised; 95% CI −3 to 2 repetitions), were non‐inferior to the partly‐supervised group.

**Conclusion:**

Remotely‐supported exercise might be an alternative to partly‐supervised exercise regarding functional fitness (assessed by timed up and go) and body composition (assessed by percentage body fat). It remains inconclusive whether remotely‐supported exercise is an alternative regarding $\dot{V}O_{2}\max$, blood pressure and other functional fitness measurements (6‐min walk, sit to stand).

**Trials Registration:** NCT06376578 (20/11/2020).

**Abbreviations**
6MWT: 6‐min walk test
BMD: bone mineral density
DBP: diastolic blood pressure
DEXA: dual‐energy X‐ray absorptiometry
ER: oestrogen receptor
HER2: human epidermal growth receptor‐2
PACES: physical activity enjoyment scale
RPE: rating of perceived exertion
SBP: systolic blood pressure
TUG: timed up and go

## 1 Introduction

In 2022 there were around 2.3 million new cases of female breast cancer diagnosed worldwide [1]. Around 55,000 cases of female breast cancer are diagnosed each year in the UK and around 77% of these women survive for more than 10 years [2]. Following diagnosis and treatment of breast cancer, cardiorespiratory fitness often decreases and body composition changes, with a decrease in lean mass and increase in fat mass [3, 4]. These changes alongside the cardiotoxicity of some breast cancer treatments [5], subsequently contribute to an increased risk of cardiometabolic disease [6, 7]. Observational and randomised‐controlled trials show that exercise training increases cardiorespiratory fitness and decreases fat mass in breast cancer survivors [8]. These changes subsequently have a positive effect on survival, disease recurrence and all‐cause mortality [9, 10, 11, 12]. Thus, improving cardiorespiratory fitness and limiting adiposity is crucial for breast cancer survivors.

The American Cancer Society, the American Society of Clinical Oncology, the Clinical Oncology Society of Australia and the American College of Sports Medicine have published guidelines or position stands advocating that exercise should be part of routine clinical management during and following cancer treatment [13, 14, 15, 16]. Although trained staff are required when employing supervised or remotely‐delivered exercise programmes, there are further barriers that hinder employing supervised exercise in oncology settings including staff capacity, resources, accessibility and costs [17]. Technology‐supported remotely‐delivered interventions provide a promising alternative strategy for prescribing exercise among breast cancer survivors without in‐person supervision [18]. Research collecting opinions on exercise among 586 cancer survivors demonstrated 57% would prefer to undertake unsupervised exercise [19], 80% would prefer to undertake remotely‐supported exercise and 68% would like to use an exercise ‘app’ or website for support [20].

Research has shown that alternatives to supervised exercise regimens can bring about beneficial effects, which might be influenced by the level of support that is provided. For example, a 12‐week randomised controlled trial of home‐based progressive exercise comprising walking, balance and stretching activities with weekly telephone calls increased predicted $\dot{V}O_{2}\max$ by 7.7% (*p* = 0.01) among 89 breast cancer survivors [21]. However, no changes in body composition were shown (BMI pre 27.7 ± 0.8 kg·m^−2^, post 27.4 ± 0.8 kg·m^−2^, *p* = 0.11). Conversely, despite extensive but less frequent support (fortnightly telephone calls and newsletters with optional monthly group exercise classes), 16 weeks of home‐based progressive exercise, comprising 150 min of moderate‐intensity aerobic exercise per week, combined with flexibility and resistance training twice weekly, did not change functional fitness—as assessed by the 6 min walk test (6MWT) – among 89 breast cancer survivors (pre 430.4 ± 101.0 m, post 454.2 ± 103.8 m, *p* > 0.05) [22]. However, body composition was not assessed.

Many supervised interventions are designed to be undertaken over a relatively short period to improve health and fitness quickly. For example, just 4 weeks of supervised moderate intensity cycling exercise 3 days per week for 30 min (combined with resistance exercises using major muscle groups) improved $\dot{V}O_{2}\max$ by 19% among 9 women receiving adjuvant radiotherapy, chemotherapy or hormone therapy for breast cancer [23]. However, most evidence showing that exercise interventions can improve physical functioning among women who have been diagnosed with breast cancer is from studies of 8–12 weeks in duration [14].

It has not been explored whether 8 weeks of unsupervised exercise training that is robustly supported using accelerometery, heart rate recording and an internet‐based data visualisation and feedback system combined with weekly telephone calls changes $\dot{V}O_{2}\max$, body composition and measurements of functional fitness among breast cancer survivors. Therefore, using a randomised controlled non‐inferiority design, the primary objective of this study was to examine whether 8 weeks of remotely‐supported exercise improves $\dot{V}O_{2}\max$ to a magnitude that is not meaningfully inferior to a partly‐supervised exercise intervention among female breast cancer survivors. Secondary objectives included examining the effects of exercise on blood pressure, functional fitness test performance (assessed via 6MWT, sit to stand, timed up and go (TUG)) and body composition (BMI, body fat percentage, bone mineral density (BMD) measures).

## 2 Methods

### 2.1 Participants

**FIGURE 1:**
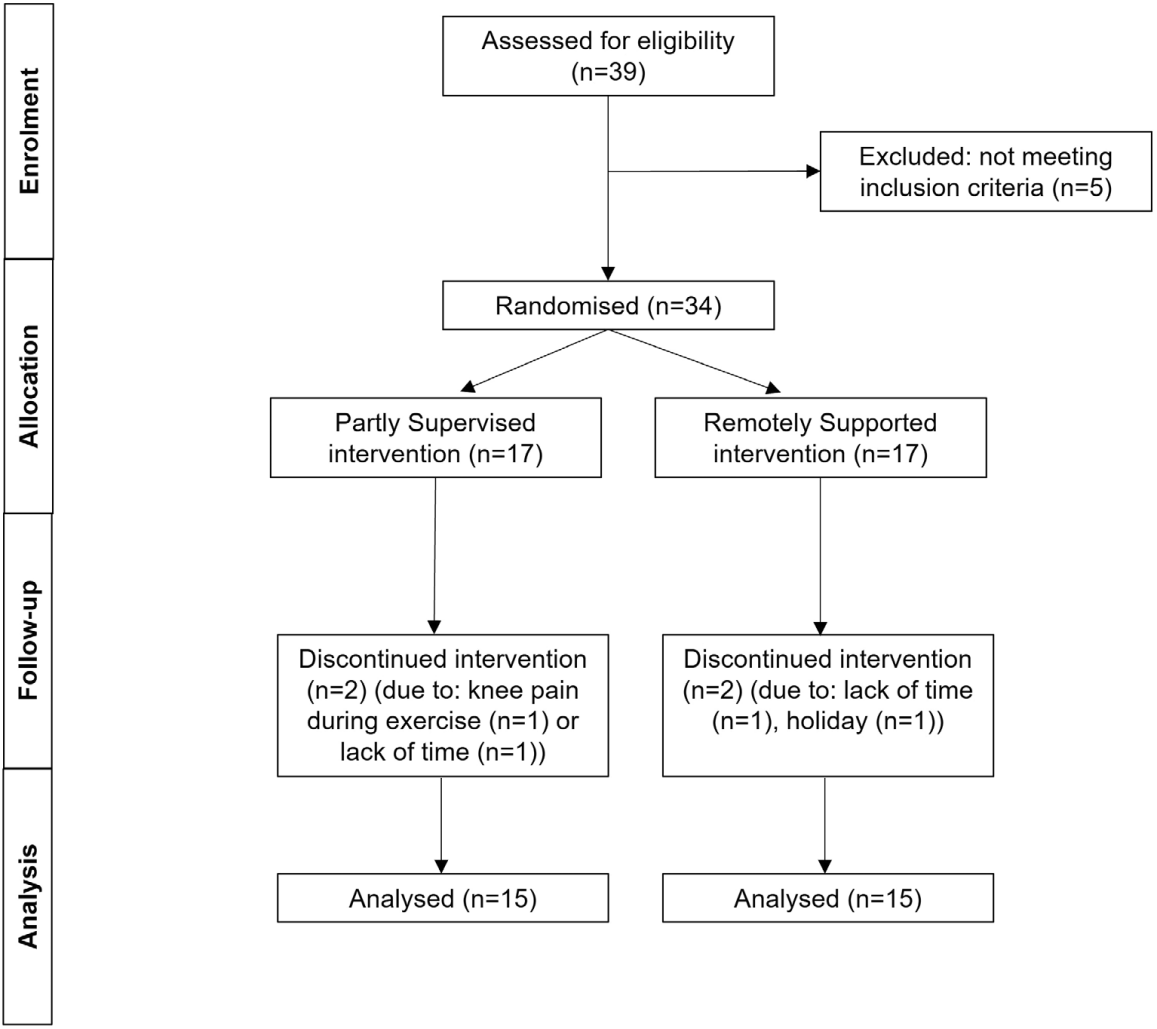
CONSORT diagram.

Thirty‐four participants were recruited between December 2018 and October 2019 at NHS Royal United Hospital, Bath. Four participants withdrew very early in the intervention and 30 participants (57 ± 6 years, BMI 25.3 ± 3.3 kg·m^−2^) were included in analyses (Figure 1). Participants were female breast cancer survivors who had received their last curative treatment (radiotherapy, chemotherapy, hormone therapy and/or surgery) at least 2 months before enrolment (i.e., to avoid immediate post‐treatment fatigue) and no longer than 5 years before enrolment (i.e., all participants were within the increased 5‐year recurrence risk window). Women on long‐term endocrine therapy were eligible. Inclusion criteria were female, 35–69 years, previous diagnosis of non‐metastatic non‐bilateral stage I–III invasive breast cancer, BMI 20–35 kg·m^−2^ and post‐menopausal (or not having had a menstrual period for at least 1 year) (Table 1). Exclusion criteria included severe hypertension (> 200/120 mmHg), cardiovascular disease, previous other malignancy except for superficial basal cell carcinoma or carcinoma‐in situ and habitually undertaking at least 30 min of self‐reported regular structured exercise on two or more occasions per week. Previous breast cancer diagnosis, oestrogen receptor (ER) and human epidermal growth receptor‐2 (HER2) status were identified from medical notes. Participants provided full and informed consent, and the study was approved by an NHS research ethics committee (reference: 18/WA/0314) in accordance with the *Declaration of Helsinki*.

**TABLE 1:** Participant characteristics at baseline. Data shown as means ± SD.

	Partly‐supervised group (*n* = 15)	Remotely‐supported group (*n* = 15)
Age (y)	58 ± 7	56 ± 6
Diagnosis		
Time since diagnosis (months)	12 ± 4	14 ± 8
ER positive	11/15 (73%)	10/15 (67%)
HER2 positive	1/15 (7%)	2/15 (13%)
Ductal carcinoma in situ	2/15 (13%)	4/15 (27%)
Multifocal carcinoma	2/15 (13%)	0/15 (0%)
Tumour Grade (G)^a^	7% (G1), 53% (G2), 27% (G3)	0% (G1), 60% (G2), 27% (G3)
TNM scoring		
Tumour (T)^a^	47% (T1), 33% (T2), 7% (T3)	47% (T1), 20% (T2), 20% (T3)
Nodes (N)	73% (N0), 27% (N1)	60% (N0), 27% (N1), 13% (N2), 0% (N3)
Metastasis (M)	100% M0	100% M0
Treatment		
Surgery	13/15 (87%)	10/15 (67%)
Time since surgery (months)	10 ± 4	12 ± 6
Chemotherapy treated	5/15 (33%)	4/15 (27%)
Radiotherapy	12/15 (80%)	10/15 (67%)
Hormone therapy	8/15 (53%)	7/15 (47%)
Anti‐HER2+ therapy	2/15 (13%)	1/15 (7%)

*Note:* Grade refers to the histologic grade (G1, G2, G3, etc.). TNM staging defines the characteristics of the tumour 0 to 4 (T) and 0 to 3 (N).

Abbreviations: ER; oestrogen receptor, HER2; Human epidermal growth factor receptor.

^a^ When total percentages do not equal 100%, DCIS staging is carcinoma in situ with no grading.

### 2.2 Sample Size

The participant recruitment target was informed by practical and logistical considerations in combination with an estimate of adequate sample size using magnitude of effect data from a similar study that examined change in V̇O_2_peak among patients with lymphoma (mean age 53 years) [24]. In this study, a 12‐week supervised exercise programme (3 sessions per week at 60%–75% V̇O_2_peak for 15–45 min), increased V̇O_2_peak (mean change 4.6 ± 3.5 mL·kg^−1^·min^−1^, 95% CI, 3.7 to 5.5 mL·kg^−1^·min^−1^). Using SampSize (https://app.sampsize.org.uk/) [25], it was estimated that for a parallel design non‐inferiority trial with 80% power and one‐sided α = 0.05, a total sample size of 28 participants (14 people per group) was needed.

### 2.3 Study Design

In this randomised controlled non‐inferiority trial, participants were randomised to one of two exercise intervention groups (allocation ratio 1:1, block size 6). The exercise intervention groups were either an 8‐week partly‐supervised group (*n* = 15), or an 8‐week remotely‐supported group (*n* = 15) (Figure 1). Given the nature of exercise interventions (i.e., in the present study, both the participant and researcher knew the group allocation by the type of exercise that was prescribed), participants, researchers nor assessors were blinded. A randomisation list was generated by one co‐author (JAB) and stratified for previous chemotherapy treatment (yes/no) and BMI (< 25 kg·m^−2^ and > 25 kg·m^−2^). An independent researcher (MT) implemented the random allocation sequence.

### 2.4 Participant Characterisation

Participants attended a laboratory for characterisation before and after the intervention (within 7 days) following a 10 h overnight fast and after refraining from exercise, alcohol and caffeine in the prior 24 h. Body mass and height were measured with the participant wearing light clothing (Tanita InnerScan Body composition monitor BC‐543; Leicester height measure, Seca Ltd). Dual‐energy X‐ray absorptiometry (DEXA) was used to quantify fat mass, lean mass and BMD parameters (Hologic Discovery W). Seated resting blood pressure was measured after 5 min of rest using an automated sphygmomanometer (Bosch and Sohn, Germany). Blood pressure was measured 3 times and averaged [26].

Three functional fitness tests were undertaken. First, the 6MWT, whereby participants walked as far as possible in 6 min between two cones placed 7 m apart [27]. Second, the sit to stand test, whereby participants performed as many sit‐to‐stands as possible in 30 s (i.e., seated on a standardised chair, rising to reach full knee extension, returning to a seated position, arms folded across the chest) [28]. Third, the TUG test, whereby participants rose from being seated on a standardised chair, walked 8 ft (2.44 m), returning to a seated position as quickly as possible [29].

Cardiorespiratory fitness was assessed using a treadmill‐based maximal walking exercise test to exhaustion (HP Cosmos Saturn, Nußdorf, Germany) based on previous protocols [30, 31]. The exercise test comprised 3 min stages, beginning at 2.7 km/h with a 1% gradient and increasing by 1.3 km/h until 6.6 km/h, with further intensity increments via increasing gradient by 2%. During the final minute of each stage, heart rate was measured using telemetry (Polar chest worn heart rate monitor RS400, Kempele, Finland) and rating of perceived exertion (RPE) was recorded [32]. Expired air samples were collected using Douglas bags during the final minute of each stage. Oxygen and carbon dioxide within each bag were analysed using a gas analyser (Servomex Group Ltd., Jarvis Brook, UK) and volume and temperature of the air were assessed using a digital thermometer and dry gas meter (Harvard Bioscience Inc., UK).

### 2.5 Intervention Groups

#### 2.5.1 Remotely‐Supported Exercise Group

The remotely‐supported group received a target for a total duration of exercise each week (outdoor walking) progressing from 105 to 150 min and 55% to 70% of $\dot{V}O_{2}\max$. By week 7, the exercise prescription aligned with common physical activity recommendations (Table 2). Participants were advised how they could break down their target into manageable bouts (e.g., 3 × 35 min walks = 105 min in week 1) and were asked to accumulate exercise with a minimum bout‐length of 10 min. Intensity was checked by participants using heart‐rate thresholds that corresponded to their $\dot{V}O_{2}\max$. Participants took part in a weekly 30 min telephone call to discuss the exercise they completed, as documented by an internet‐based data visualisation platform with data input from a wrist worn fitness tracker (Polar A370, Polar Electro, Kempele, Finland) that recorded accelerometery data and heart rate via photoplethysmography. During this telephone call, researchers discussed whether participants had met the recommended duration and intensity of prescribed exercise by interpreting the accelerometery output and comparing heart rate measurements to heart rate thresholds that corresponded to the prescribed intensity expressed as percentages of $\dot{V}O_{2}\max$.

**TABLE 2:** Exercise prescription.

	Week 1 & 2	Week 3 & 4	Week 5 & 6	Week 7 & 8
Partly‐supervised group				
Intensity (% $\dot{V}O_{2}\max$)	55	60	65	70
Duration (min)	105	120	135	150
Duration breakdown				
Treadmill (min)	20	25	30	35
Bike (min)	15	15	15	15
Total after 1 session (min)	35	40	45	50
Total after 2 sessions week (min)	70	80	90	100
Unsupervised walking (min)	35	40	45	50
Remotely‐supported group				
Intensity (% $\dot{V}O_{2}\max$)	55	60	65	70
Duration (min)	105	120	135	150
Advice for duration breakdown				
Simplest breakdown (bouts × min)	3 × 35	3 × 40	3 × 45	3 × 50
Minimum bout‐length (bouts × min)	≈11 × 10	12 × 10	≈14 × 10	15 × 10

Abbreviations: Min, minutes; $\dot{V}O_{2}\max$; maximum oxygen update.

#### 2.5.2 Partly‐Supervised Exercise Group

The partly‐supervised exercise group undertook 2 supervised (laboratory‐based treadmill and cycle ergometer exercise) exercise sessions progressing from 35 to 50 min and 55% to 70% of $\dot{V}O_{2}\max$ and 1 unsupervised session per week (e.g., outdoor walking). By week 7, the exercise prescription aligned with common physical activity recommendations (Table 2). During supervised laboratory sessions during treadmill walking, 1 min of expired air was collected into Douglas bags after 15, 25, 35 min (depending on intervention week) and heart rate was recorded. If VO_2_, did not match the prescribed exercise intensity then the gradient and speed was altered accordingly, and a subsequent expired air collection was undertaken. During cycling exercise, an almost identical process was undertaken after 5 min of exercise. The intensity of unsupervised exercise sessions was recorded using a chest‐worn heart rate monitor (Wahoo Fitness, Atlanta, Georgia, USA).

### 2.6 Intervention Adherence and Enjoyment

In the remotely‐supported group, adherence was assessed by participant verbal confirmation of reaching the weekly exercise target (duration and intensity) verified via data interpretation from wrist‐worn heart rate recordings and accelerometery (Polar A370 fitness tracker, Polar Electro, Kempele, Finland). In the partly‐supervised group, adherence was assessed by completion of each supervised session (via duration recording) and participants' verbal confirmation of completing each unsupervised session, verified via data interpretation of chest‐worn heart‐rate recordings (Wahoo Fitness, Atlanta, Georgia, USA). The physical activity enjoyment scale (PACES) was administered 4 weeks into the intervention to assess enjoyment [33] among both groups. Participants were asked to rate “how you feel at the moment about the physical activity you have been doing” using a 7‐point Likert scale from 1 (I enjoy it) to 7 (I hate it). Eleven items were worded negatively (and reverse scored); seven items were worded/scored positively. Scores can range from 18 to 126, with higher scores indicating higher enjoyment.

### 2.7 Statistical Analysis

One‐way ANOVAs and Mann–Whitney U tests were employed to test differences in adherence and PACES data between groups. For non‐inferiority analyses, a point estimate method was used whereby the largest clinically and/or physiologically meaningful difference between the test (partly‐supervised exercise group) and the active comparator (remotely‐supported exercise group) was defined (i.e., Δ see Table 3). When the difference between groups was smaller than this difference, the active comparator (remotely‐supported exercise group) was considered to elicit a change that was clinically and/or physiologically non‐inferior in magnitude compared to the test (partly‐supervised exercise group). Two‐sided 95% CIs between the difference of treatments were calculated by calculating the variance of the difference between groups $\sqrt{\frac{\mathrm{SD}\left(\mathrm{PS}\right)}{n\left(\mathrm{PS}\right)}+\frac{\mathrm{SD}\left(\mathrm{RS}\right)}{n\left(\mathrm{RS}\right)}}$, whereby *SD(PS; partly‐supervised)* represents the standard deviation of the change within the partly‐supervised group, *SD(RS; remotely‐supported)* represents the standard deviation of the change within the remotely‐supported group, *n(PS; partly‐supervised)* represents the number of participants in the partly‐supervised group and *n(RS; remotely‐supported)* represents the number of participants in the remotely‐supported group. The variance was then multiplied by the constant Z‐score (1.96) and subsequently added or subtracted from the average difference in the means between the partly‐supervised group and the remotely‐supported group to calculate the upper and lower CIs. The following criteria were defined based on the upper and lower limits (1) If the CI lies wholly above/below (whatever was deemed worse) 0 and Δ, the remotely‐supported group is non‐inferior to the partly‐supervised group, (2) If the CI lies above/below Δ (whatever was deemed worse) but crosses 0, the remotely‐supported group is non‐inferior to the partly‐supervised group, (3) If the CI lies above/below 0 (whatever was deemed beneficial) but includes Δ, the results are inconclusive and (4) If the CI lies above/below 0 (whatever was deemed beneficial) and Δ the remotely‐supported is inferior to the partly‐supervised group [45]. To aid data interpretation, difference‐based analysis was conducted using repeated measures two‐way ANOVAs and separate one‐way ANOVAs. Statistical analyses were conducted using SPSS version 22. Figures were produced using GraphPad Prism 8.

**TABLE 3:** Participant characteristics at baseline and the largest clinically or physiologically meaningful differences.

	Partly‐supervised group (*n* = 15)	Remotely‐supported group (*n* = 15)	Largest meaningful difference (Δ)	Supporting reference for Δ
$\dot{V}O_{2}\max$ and blood pressure				
$\dot{V}O_{2}\max$ (mL·kg ^−1^·min^−1^)	28.3 ± 3.9	28.9 ± 6.8	3.0	[34]
Systolic BP (mmHg)	135 ± 22	125 ± 21	2	[35, 36]
Diastolic BP (mmHg)	80 ± 8	80 ± 11	1	[35, 36]
Functional fitness				
6‐min walk (m)	475 ± 43	493 ± 72	14	[37]
Sit to stand (reps)	16 ± 4	16 ± 4	2	[38]
Get up and go (s)	5.0 ± 0.8	5.0 ± 1	2.1	[39]
Body composition				
BMI (kg·m^−2^)	25.8 ± 2.9	24.4 ± 3.4	3.0	[40]
Body fat (%)	37.0 ± 3.9	35.2 ± 7.3	5.0	[41]
Lean mass (kg)	41.7 ± 4.3	42.2 ± 3.8	5.0	[42]
BMD (g·cm^−2^)	1.13 ± 0.11	1.10 ± 0.08	3.0	[43]
Z‐score	0.4 ± 1.1	0.2 ± 0.7	2.0	[44]
T‐score	0.2 ± 1.3	−0.1 ± 1.0	1.0	[44]
Adherence and enjoyment				
Sessions completed (%)	87 ± 7	64 ± 25*	—	—
Enjoyment	94 ± 14	80 ± 13*	—	—

*Note:* Data shown as means ± SD. A change that is non‐inferior in magnitude or direction (Δ) between the test (partly‐supervised group) and the active comparator (remotely‐supported group). ‐ indicates data were not included in non‐inferiority analysis.

Abbreviations: $\dot{V}O_{2}\max$, maximum oxygen update; BP; blood pressure; cm; centimetres; g; grams; kg, kilogramme; m; metres; min, minutes; mmHg; millimetres of mercury; s; seconds.

* Significantly different between groups, *p* < 0.05.

## 3 Results

Adherence was significantly higher in the partly‐supervised group (87% ± 7%) compared to the remotely‐supported group (64% ± 25%) (*F*_[1,28]_ = 9.923, *p* = 0.004, effect size 0.26). However, in both groups, all sessions were attempted by all participants (i.e., no sessions were missed), therefore the lack of adherence reflects failure to achieve the prescribed duration and/or intensity of affected sessions (see Table S1). Enjoyment was significantly higher in the partly‐supervised group (94 ± 14) compared to the remotely‐supported group (80 ± 13) (*U* = 51.000, *p* = 0.010, effect size 0.47). The remaining results are presented as a non‐inferiority analysis, but to help with data interpretation, a traditional difference‐based analysis is summarised in the final part of the results section and shown in the additional information (see Tables S2 and S3).

### 3.1 Cardiorespiratory Fitness and Blood Pressure

**FIGURE 2:**
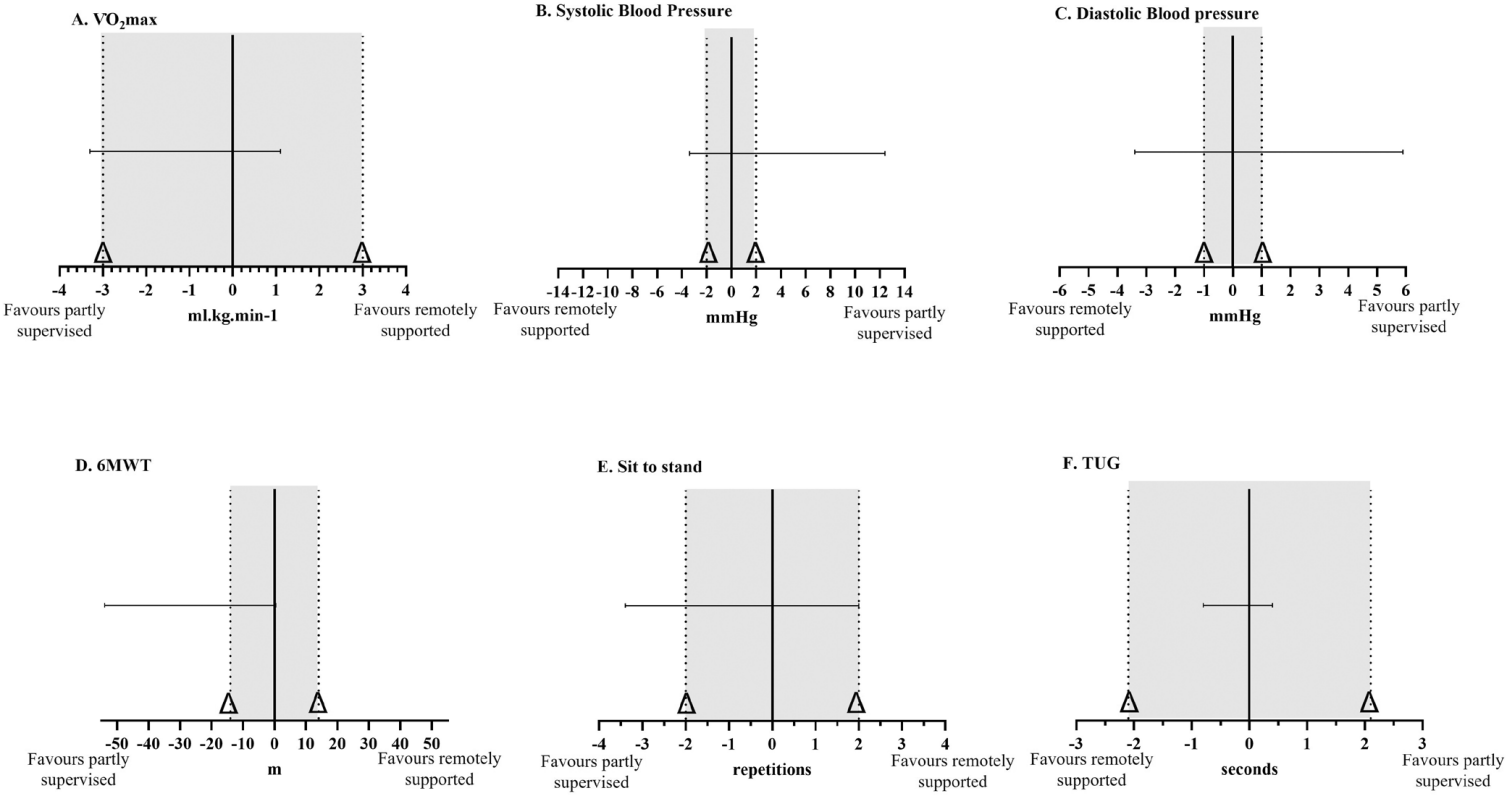
Non‐inferiority analysis of the remotely‐supported group compared to the partly‐supervised group. (A) $\dot{V}O_{2}\max$, (B) Systolic blood pressure, (C) diastolic blood pressure. (D) 6MWT (6‐min walk test), (E) sit to stand, (F) $\dot{V}O_{2}\max$, maximum oxygen uptake; kg; kilogramme; m; metres; min, minute; mL, millilitre; mmHg, millimetres of mercury; TUG, timed up and g; Δ, margin. Dotted line represents the non‐inferiority margin (Δ).

Change in $\dot{V}O_{2}\max$ in the remotely‐supported group was −0.74 ± 2.21 mL·kg^−1^·min^−1^ (95% CI −2.0 to 0.48 mL·kg^−1^·min^−1^) compared to the partly‐supervised group which was +0.35 ± 3.50 mL·kg^−1^·min^−1^ (95% CI −1.59 to 2.28 mL·kg^−1^·min^−1^). The 2‐sided 95% CI for the difference between groups was −3.3 to 1.1 mL·kg^−1^·min^−1^. The lower limit of the CI (−3.3 mL·kg^−1^·min^−1^) is lower than the non‐inferiority margin (−3.0 mL·kg^−1^·min^−1^) and the 95% CI for the difference between groups crosses 0. Given that higher $\dot{V}O_{2}\max$ is beneficial, it is inconclusive whether the change among the remotely‐supported group (Pre: 28.9 ± 6.8 mL·kg^−1^·min^−1^, Post 28.2 ± 7.1 mL·kg^−1^·min^−1^) was non‐inferior in magnitude to the change exhibited by the partly‐supervised group (Pre: 28.2 ± 3.9 mL·kg^−1^·min^−1^, Post: 28.7 ± 4.8 mL·kg^−1^·min^−1^) (Figure 2A).

Change in systolic blood pressure (SBP) in the remotely‐supported group was −5 ± 11 mmHg (95% CI −12 to 2 mmHg) compared to the partly‐supervised group which was −9 ± 10 mmHg (95% CI −14 to −4 mmHg). The 2‐sided 95% CI for the difference between groups was −3 to 12 mmHg. The upper limit of the CI (12 mmHg) is greater than the non‐inferiority margin (2 mmHg), and the 95% CI for the difference between groups crosses 0. Given that lower SBP is beneficial, it cannot be concluded that the change among the remotely‐supported group (Pre: 125 ± 21 mmHg, Post: 121 ± 19 mmHg) was non‐inferior in magnitude to the change exhibited by the partly‐supervised group (Pre: 135 ± 22 mmHg, Post: 126 ± 22 mmHg) (Figure 2B).

Change in diastolic blood pressure (DBP) in the remotely‐supported group was −1 ± 6 mmHg (95% CI −5 to 2 mmHg) compared to the partly‐supervised group which was −2 ± 8 mmHg (95% CI −6 to −3 mmHg). The 2‐sided 95% CI for the difference between groups was −5 to 6 mmHg. The upper limit of the CI (6 mmHg) is greater than the non‐inferiority margin (1 mmHg), and the 95% CI for the difference between groups crosses 0. Given that a lower diastolic blood pressure is beneficial, it cannot be concluded that the change among the remotely‐supported group (Pre: 80 ± 11 mmHg, Post: 79 ± 13 mmHg) was non‐inferior in magnitude to the change exhibited by the partly‐supervised group (Pre: 80 ± 8 mmHg, Post: 79 ± 10 mmHg) (Figure 2C).

### 3.2 Functional Fitness

Change in 6MWT distance in the remotely‐supported group was +15.7 ± 35.9 m (95% CI −4.1 to +35.6 m) compared to the partly‐supervised group which was +42.5 ± 36.7 m (95% CI 22.2 to 62.8 m). The 2‐sided 95% CI for the difference between groups was −54.0 to 0.4 m. The lower limit of the CI (−54 m) is lower than the non‐inferiority margin (−14 m), and the 95% CI for the difference between groups crosses 0. Given that a further 6MWT distance is better, it is inconclusive whether the change among the remotely‐supported group (Pre: 493.2 ± 73.1 m, Post: 508.9 ± 76.1 m) was non‐inferior in magnitude to the change exhibited by the partly‐supervised group (Pre: 475.1 ± 42.5 m, Post: 517.6 ± 35.3 m) (Figure 2D).

Change in sit to stand repetitions in the remotely‐supported group was +2 ± 3 repetitions (95% CI 0 to 4 repetitions) compared to the partly‐supervised group which was +3 ± 4 repetitions (95% CI 1 to 5 repetitions). The 2‐sided 95% CI for the difference between groups was −3 to 2 repetitions. The lower limit of the CI (3 repetitions) is lower than the non‐inferiority margin (2 repetitions) and the 95% CI for the difference between groups crosses 0. Given that higher sit to stand repetitions is beneficial, it cannot be concluded that the change among the remotely‐supported group (Pre: 16 ± 4, Post: 18 ± 5 repetitions) was non‐inferior in magnitude to the change exhibited by the partly‐supervised group (Pre: 16 ± 4, Post: 19 ± 5 repetitions) (Figure 2E).

Change in TUG time in the remotely‐supported group was −0.4 ± 0.8 s (95% CI −0.9 to 0 s) compared to the partly‐supervised group which was −0.2 ± 0.8 s (95% CI −0.7 to 0.2 s). The 2‐sided 95% CI for the difference between groups was −0.8 to 0.4 s. The upper limit of the CI (0.4 s) is lower than the non‐inferiority margin (2.1 s). Given that a lower TUG time is beneficial, change among the remotely‐supported group (Pre: 5.0 ± 1.0 s, Post: 5.0 ± 1.0 s) was considered to be non‐inferior in magnitude to the partly‐supervised group (Pre: 5.0 ± 0.8 s, Post: 4.8 ± 0.8 s) (Figure 2F).

### 3.3 Body Composition

**FIGURE 3:**
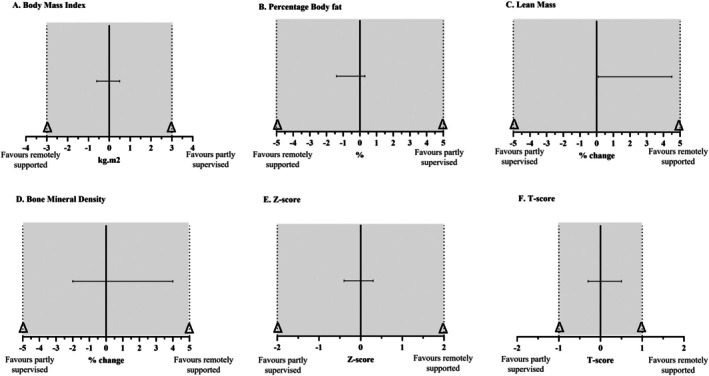
Non‐inferiority analysis of the remotely‐supported group compared to the partly‐supervised group. (A) Body mass index; (B) percentage body fat, (C) lean mass, (D) bone mineral density, (E) Z‐score, (F) T‐score. kg, kilogramme; m, metres; min, minute; Δ, margin. Dotted line represents the non‐inferiority margin (Δ).

Change in BMI in the remotely‐supported group was 0.0 ± 0.6 kg·m^−2^ (95% CI −0.3 to 0.4 kg·m^−2^) compared to the partly‐supervised group which was +0.1 ± 0.9 kg·m^−2^ (95% CI −0.4 to 0.6 kg·m^−2^). The 2‐sided 95% CI for the difference between groups is −0.6 to 0.5 kg·m^−2^. The upper limit of the CI (kg·m^−2^) is less than the non‐inferiority margin (3.0 kg·m^−2^). Given that a lower BMI is generally beneficial, the change in BMI among the remotely‐supported group (Pre: 24.4 ± 3.4 kg·m^−2^, Post: 24.4 ± 3.5 kg·m^−2^) was considered to be non‐inferior in magnitude to the partly‐supervised group (Pre: 25.8 ± 2.9 kg·m^−2^, Post: 25.9 ± 3.2 kg·m^−2^) (Figure 3A).

Change in body fat percentage in the remotely‐supported group was −0.6% ± 1.3% (95% CI −1.3% to 0.2%) compared to the partly‐supervised group which was −0.1% ± 1.1% (95% CI −0.6% to 0.5%). The 2‐sided 95% CI for the difference between groups was −1.4% to 0.3%. The upper limit of the CI (0.3%) is less than the non‐inferiority margin (5.0%). Given that a lower body fat percentage is generally beneficial, the change among the remotely‐supported group (Pre: 35.2% ± 7.3%, Post: 34.6% ± 7.6%) was considered to be non‐inferior in magnitude to the partly supervised group (Pre: 37.0% ± 3.9%, Post: 37.0% ± 3.6%) (Figure 3B).

Change in lean mass percentage in the remotely‐supported group was +1.0% ± 2.0% (95% CI −0.2% to 2.1%) compared to the partly‐supervised group which was −1.3% ± 3.6% (95% CI −0.9% to 1.7%). The 2‐sided 95% CI for the difference between groups was +0.1% to 4.5%. The lower limit of the CI (0.1%) is less than the non‐inferiority margin (5%). Given that a higher lean mass is beneficial, the change among the remotely‐supported group (Pre: 42.2 ± 3.8 kg, Post: 41.6 ± 3.8 kg) was considered to be non‐inferior in magnitude to the partly‐supervised group (Pre: 41.7 ± 4.3 kg, Post: 41.9 ± 4.3 kg) (Figure 3C).

Change in BMD in the remotely‐supported group was −1% ± 4% (95% CI −3 to 1) compared to the partly‐supervised group which was 0% ± 3% (95% CI −2% to 1%). The 2‐sided 95% CI for the difference between groups was −2% to 4%. The lower limit of the CI (−2%) is greater than the non‐inferiority margin (−5%). Given that a higher BMD is beneficial, change among the remotely‐supported group (Pre: 1.098 ± 0.078 g·cm^−3^, Post: 1.096 ± 0.085 g·cm^−3^) was considered to be non‐inferior in magnitude to the partly‐supervised group (Pre: 1.128 ± 0.106 g·cm^−3^, Post: 1.112 ± 0.100 g·cm^−3^) (Figure 3D).

Change in BMD Z‐score in the remotely‐supported group was −0.13 ± 0.4 (95% CI −0.37 to 0.11) compared to the partly‐supervised group which was −0.05 ± 0.5 (95% CI −0.30 to 0.19). The 2‐sided 95% CI for the difference between groups was −0.4 to 0.3. The lower limit of the CI (−0.4) is more negative than the non‐inferiority margin (−2.0). Given that a higher Z‐score is beneficial, change among the remotely‐supported group (Pre: 0.2 ± 0.7, Post: 0.1 ± 0.7) was considered to be non‐inferior in magnitude to the partly‐supervised group (Pre: 0.4 ± 1.1, Post: 0.3 ± 0.9) (Figure 3E).

Change in BMD T‐score in the remotely‐supported group was −0.06 ± 0.5 (95% CI −0.31 to 0.19) compared to the partly‐supervised group which was −0.16 ± 0.5 (95% CI −0.43 to 0.11). The 2‐sided 95% CI for the difference between groups was −0.3 to 0.5. The lower limit of the CI (−0.3) is more negative than the non‐inferiority margin (−1.0). Given that a higher T‐score is beneficial, change among the remotely‐supported group (Pre: −0.1 ± 1.0, Post: −0.1 ± 0.9) was considered to be non‐inferior in magnitude to the partly‐supervised group (Pre: 0.2 ± 1.3, Post: 0.0 ± 1.2) (Figure 3F).

### 3.4 Difference‐Based Analysis

Post hoc difference‐based analysis is shown for cardiorespiratory fitness, blood pressure and functional fitness measurements in Table S2. No significant differences were shown for $\dot{V}O_{2}\max$ between pre‐ and post‐intervention in either group. A statistically significant decrease in systolic blood pressure was shown post‐intervention in the partly‐supervised group only (135 ± 22 mmHg to 126 ± 22 mmHg; *F*_(1,14)_ = 12.965, *p* = 0.003, *ηp* ^2^ = 0.23). Participants in the partly‐supervised group improved 6MWT distance by 43 m (*F*_(1,14_) = 20.106, *p* = 0.001, *ηp* ^2^ = 0.35, large effect) and performance in the sit to stand test by 3 repetitions (*F*_(1,14)_ = 7.166, *p* = 0.018, *ηp* ^2^ = 0.12, medium effect). In the remotely‐supported group, there were no statistically significant differences in 6MWT or TUG, but there was a statistically significant improvement in the number of repetitions in the sit to stand test (*F*_(1,14)_ = 6.364, *p* = 0.024, *ηp* ^2^ = 0.10, medium effect). There were no statistically significant differences in body composition pre‐ versus post‐intervention in either group (Table S3).

## 4 Discussion

This study examined whether 8 weeks of remotely‐supported exercise training improved cardiorespiratory fitness, functional fitness and body composition to a magnitude that was not meaningfully inferior to 8 weeks of partly‐supervised exercise training among female breast cancer survivors. The results demonstrate that 8 weeks of remotely‐supported exercise might be an alternative to 8 weeks of partly‐supervised exercise for improving performance in the TUG test and for influencing body composition (BMI, percentage body fat, lean mass, BMD and BMD Z‐score and T‐score). Yet it remains inconclusive whether remotely‐supported exercise is a viable alternative to partly‐supervised exercise for the effects on cardiorespiratory fitness (V̇O_2_ max), blood pressure and performance in other functional fitness tests (6MWT, sit to stand). Finally, adherence was higher in the partly‐supervised group (87% ± 7%) compared to the remotely‐supported group (64% ± 25%).

These results provide an important contribution to the literature, given that a recent systematic review concluded there is insufficient evidence to confirm whether remotely‐supported exercise interventions are useful for helping cancer survivors to become more active or improve aspects of fitness [46]. Indeed, remotely‐supported interventions allow for greater equity of care across the diverse population of breast cancer survivors and have greater scalability versus supervised exercise given the lower staffing requirements, need for facilities and travel for patients [17, 47]. However, a complexity within this research area, is that many remotely‐delivered interventions—which are often unsupervised and unsupported (unlike the present study) – are compared to interventions that are extensively supported by in‐person supervision [48]. Thus, support often differs substantially between groups *within* studies and *between* studies, influencing differences in adherence and potential change in outcomes. For example, some remotely‐supported, unsupervised interventions do not provide much support other than the initial guidance or exercise prescription (e.g., a leaflet or exercise training plan) [48], whereas some studies offer prompts and reminders (e.g., text messages, telephone calls) [21] and others provide more comprehensive support (e.g., real‐time physiological monitoring and activity tracking) [49]. In the present study, the remotely‐supported group received instantaneous feedback about exercise intensity (i.e., via heart rate) and participants could assess daily whether they had met their exercise volume target, using recorded heart rate data, accelerometery and an internet‐based data‐visualisation and feedback system supplemented with weekly telephone calls providing guidance and advice. Thus, comparisons between the present results from an exercise intervention group that was remotely‐supported in a more comprehensive way compared to other interventions that are described as being “remotely delivered” (of which many are unsupervised and unsupported) need to be interpreted considering the level of support provided.

Cardiorespiratory fitness is commonly assessed in exercise training interventions among cancer survivors. In the present study, it could not be concluded whether changes in $\dot{V}O_{2}\max$ among the remotely‐supported group (−0.74 ± 2.21 mL·kg^−1^·min^−1^ (95% CI −2.0 to 0.48 mL·kg^−1^·min^−1^)) were non‐inferior in magnitude compared to the partly‐supervised group (+0.35 ± 3.50 mL·kg^−1^·min^−1^ (95% CI −1.59 to 2.28 mL·kg^−1^·min^−1^)). This might have been influenced by the nature and frequency of support provided in the remotely‐supported group. Generally, supervised exercise training regimens among cancer survivors improve cardiorespiratory fitness. For example, a meta‐analysis of supervised exercise interventions (compared most commonly to standard care) concluded that aerobic exercise training improved V̇O_2_peak among cancer survivors (18 comparisons, 1093 participants; standardised mean pooled effect: 0.74; 95% CI: 0.52 to 0.96; *p* < 0.001) [50]. Pooling of the 13 studies reporting V̇O_2_peak showed a statistically significant mean difference of +3.13 mL·kg^−1^·min^−1^; 95% CI: 2.2 to 4.05 mL·kg^−1^·min^−1^; *p* < 0.001. Improvements in cardiorespiratory fitness have also been reported among overweight breast cancer survivors (*n* = 25, 30–55 years) following 10 weeks of supervised aerobic exercise at 40%–75% maximal heart rate [51]. However, despite divergent patterns between groups, it must be considered that for both groups in the present study, exercise training had a minimal impact on $\dot{V}O_{2}\max$, as shown by traditional difference‐based analyses. We speculate that this could be because of difficulties in measuring a true $\dot{V}O_{2}\max$ value in this population, which could have resulted in participants being prescribed exercise at a lower intensity than intended [52, 53]. Indeed, similar to the present investigation, another study examining 12 weeks of home‐based unsupervised aerobic exercise training among 10 breast cancer survivors, has also reported no change in $\dot{V}O_{2}\max$ (mean change +0.96 mL·kg^−1^·min^−1^, 95% CI −0.75 to 2.69, *p* = 0.236), despite remote heart rate monitoring and weekly contact addressing exercise intervention adherence [54]. Although it could be argued that short‐duration interventions over several weeks are less likely to change $\dot{V}O_{2}\max$, a randomised controlled trial of 6‐months exercise training among breast cancer survivors, also showed no statistically significant changes to $\dot{V}O_{2}\max$, *n* = 15 (*n* = 15 intervention group; 24.1 ± 4.8 mL·kg^−1^·min^−1^ pre‐intervention to 26.2 ± 5.3 mL·kg^−1^·min^−1^ post‐intervention, versus *n* = 14 control group; 26.6 ± 4.4 mL·kg^−1^·min^−1^ pre‐intervention to 26.6 ± 3.3 mL·kg^−1^·min^−1^ post‐intervention). The intervention consisted of an initial consultation, followed by ~monthly script‐guided telephone calls to encourage 30 min of moderate‐intensity physical activity on 3–5 days per week [55]. However, other studies, assessing fitness in less precise ways, have indicated that regular support—even if not remotely monitored—might be important for improving outcomes. A study investigating 12 weeks of home‐based unsupervised aerobic exercise training using prescribed heart rate intensities and heart rate monitoring among 10 breast cancer survivors reported no change in $\dot{V}O_{2}\max$ (mean change +0.96 mL·kg^−1^·min^−1^, 95% CI −0.75 to 2.69, *p* = 0.236), despite remote monitoring and weekly contact addressing exercise intervention adherence [54]. Conversely, another unsupervised intervention showed that 12 weeks of physical activity counselling and exercise guidance leaflets, without remote monitoring among 86 patients with breast cancer, increased self‐reported physical activity levels and fitness, measured by the Rockport 1‐mile walk test (6% reduction in time taken to complete the test, *p* < 0.001) [56]. Future research should investigate the components of a remotely‐supported exercise intervention that leads to improvements in study outcomes, and in particular $\dot{V}O_{2}\max$. Further research should consider different strategies for measuring and validating $\dot{V}O_{2}\max$ in this population (52, 53).

In the current study, there was a statistically significant change in SBP from 135 ± 22 mmHg to 126 ± 22 mmHg in the partly‐supervised group, but not in the remotely‐supported group (from 125 ± 21 mmHg to 121 ± 20 mmHg). There was a negligible change of ~1 mmHg in DBP among both groups. As such, the change in SBP among the remotely‐supported group could not be considered as being non‐inferior in magnitude to the partly‐supervised group. A similar reduction in SBP as shown in the partly‐supervised group has been reported previously in a randomised controlled supervised exercise intervention trial among 26 breast cancer survivors 2–6 months after chemotherapy. SBP decreased by 6 mmHg (95% CI 4 to 8 mmHg) following an 8‐week aerobic exercise intervention (*n* = 16, 50 ± 8 years) compared to a reduction of 2 mmHg (95% CI 0 to 5 mmHg) in a non‐exercising control group (*n* = 10, 45 ± 9 years). The exercise intervention consisted of twice weekly supervised exercise sessions ranging from 35%–65% heart rate reserve, progressing from 21 to 42 min employing 3 modes of stationary exercise (cycling, walking, rowing) [57]. However, in a study of 177 patients with breast cancer (50 ± 10 years, 23.3 ± 3.3 kg·m^−2^) following completion of primary treatment, a progressive 12 week home‐based moderate intensity aerobic and resistance training exercise intervention elicited no significant differences in SBP post intervention (119 ± 18 mmHg to 118 ± 17 mmHg), probably due to “normal” SBP values prior to the intervention [58]. Likewise, in the current study, the blood pressure profile of each intervention group likely influenced the results (i.e., baseline SBP among the partly‐supervised group was closer to “high normal” at 135 ± 22 mmHg compared to 125 ± 21 mmHg among the remotely‐supported group; although differences between groups were not statistically significant). Due to the increased risk of cardiovascular events with higher blood pressure [59], and the increased risk of cardiovascular disease in women who have undergone breast cancer treatment [60], interventions that reduce blood pressure may be particularly important for breast cancer survivors.

Breast cancer survivors typically self‐report lower levels of functional fitness compared to women without cancer [61, 62]. Thus, objective functional fitness measurements may better estimate the true prevalence of functional limitations among breast cancer survivors [63]. In the present study, it could not be concluded that the change in 6MWT performance in the remotely‐supported group was non‐inferior in magnitude compared to the partly‐supervised group. Further, traditional difference‐based analysis (see Table S2) showed that only the partly‐supervised intervention elicited a statistically significant change in 6MWT performance, which improved by +43 ± 37 m versus a non‐significant change of +17 ± 36 m in the remotely‐supported group. A systematic review examining 6 exercise intervention studies among adults with medical conditions including cardiovascular and respiratory disease (*n* = 364, interventions 7–12 weeks) concluded that improvements in 6MWT performance over 14 m should be considered clinically meaningful [37]. Indeed, the change in 6MWT performance in the present study could indicate that 8 weeks of *either* partly‐supervised or remotely‐supported exercise training might reduce cardiovascular disease risk among breast cancer survivors [64]. Other remotely‐supported exercise interventions have reported similar results to the present study. For example, 13 female breast cancer survivors (58 ± 3 years, 25.7 ± 0.6 kg·m^−2^) participated in an 8‐week group tele‐exercise intervention. Exercise sessions were 60 min, took place twice weekly and comprised aerobic and resistance exercise, supervised via video‐calls. 6MWT performance increased by 47 m, from 495 ± 13 m at baseline to 542 ± 14 m post‐ intervention (*p* < 0.001) [65]. This change is similar to the present study (remotely‐supported group; increase of 17 ± 36 m from 493 ± 72 m at baseline to 510 ± 76 m after 8 weeks; partly‐supervised group; increase of 43 ± 37 m, 475 ± 43 m at baseline to 518 ± 35 m after 8 weeks). However, these results should be interpreted with caution given the influence that walking track length has on results. In the present study, due to practical limitations, the walk test track was 7 m, when most studies use 10, 20 or 30 m, and a shorter track typically results in shorter distances achieved due to the number of turns [66]. However, not all remotely‐supported exercise training interventions have reported changes in 6MWT performance among breast cancer survivors. For example, one study examined 12 weeks of unsupervised home‐based exercise, comprising 10 min of body‐weight based high intensity interval training three times per week, among *n* = 50 women with stage I–IIa breast cancer, 20–59 years, who had completed initial treatment except for hormone therapy. The results showed no changes in 6MWT performance (Pre: 586 ± 43 m, Post: 603 ± 59 m, *p* = 0.93) [67]. Failure to improve 6MWT performance might have been influenced by the relatively short bouts of exercise in each prescribed session, or the nature of support provided, which comprised personalised e‐mails and a smart‐phone app, rather than person‐delivered feedback and guidance (e.g., telephone calls as with the present study). Such factors—in particular exercise prescription characteristics and support provided—which appear to influence the effectiveness of interventions, need to be considered when applying the findings of the present study and others cited here to public health recommendations.

The results of this study demonstrate that 8 weeks of remotely‐supported exercise among breast cancer survivors might be an alternative to 8 weeks of partly‐supervised exercise given the effects on functional fitness, when assessed by the TUG test. However, difference‐based analysis showed that neither intervention caused a statistically significant change in TUG performance (remotely‐supported group pre‐intervention 5.0 ± 1.1 s, post‐intervention 5.0 ± 1.1 s; partly‐supervised group pre‐intervention 5.0 ± 0.8 s, post‐intervention 4.8 ± 0.8 s). Indeed, the smallest clinically meaningful difference in TUG performance is 2.1 s [39]. Improvements in TUG performance have been shown following longer duration (12‐week) interventions which incorporate resistance exercise [68]. In the present study, change in sit to stand test score in the remotely‐supported group could not be concluded as being non‐inferior in magnitude compared to the partly‐supervised group. Further, traditional difference‐based analysis (see Table S2) showed that only the partly‐supervised intervention elicited a statistically significant change in sit to stand test score, which improved by +3 ± 4 repetitions versus a non‐statistically significant change of +2 ± 3 repetitions in the remotely‐supported group. However, it is worth emphasising that improvements in sit to stand test score over 2 repetitions have been considered as being clinically meaningful [37]. Therefore, the change in sit to stand test score in the present study could indicate that 8 weeks of *either* partly‐supervised or remotely‐supported exercise training might improve functional fitness. This is encouraging given that even a longer duration, 16‐week home‐based exercise intervention among 89 women following breast cancer treatment did not improve sit to stand score compared to a control group [69].

Given the links between obesity and breast cancer recurrence, or more broadly, cardiometabolic disease and overall mortality, many exercise training interventions among cancer survivors aim to change body composition. In the present study, it was concluded that change in aspects of body composition among the remotely‐supported group was non‐inferior in magnitude compared to the partly‐supervised group. However, it must be considered that across both groups, exercise training had minimal impact on body composition, as shown by traditional difference‐based analyses (see Table S3). These effects could have been influenced by participants not being obese at baseline, thus having less “weight” to lose (BMI: partly‐supervised group: 25.8 ± 2.9 kg·m^−2^, remotely‐supported group: 24.4 ± 3.4 kg·m^−2^). These group characteristics might be a consequence of selection bias—those willing to engage in exercise following breast cancer treatment were likely more motivated to participate in the study. However, the lack of change in body composition could also be due to the aerobic design of the present interventions, given that many studies reporting changes to body composition employ combined exercise and diet interventions [70] or multi‐modal exercise including both aerobic and resistance exercise. Indeed, in the present study, aerobic exercise without resistance exercise, over a relatively short intervention period of 8 weeks, may not be optimal for increasing lean mass and decreasing fat mass [71]. For example, in the current study, change in lean mass percentage was small (remotely‐supported group: 1.0% ± 2.0%, 95% CI −0.2% to 2.1%, partly‐supervised group: −1.3% ± 3.6%, 95% CI −0.9% to 1.7%; also see Table S3). Further research should investigate the efficacy of combined resistance and aerobic exercise in a remotely‐supported format to explore whether body composition is affected. An alternative explanation for a lack of change to body composition could be a compensatory eating response [72]. Inconsistent effects of exercise interventions on body composition are often reported *within* and *between* studies. A systematic review of systematic reviews assessed the effectiveness of supervised and unsupervised lifestyle interventions (e.g., ranging in duration from 2 weeks to 24 months) among female breast cancer survivors for changing body composition. Eight of 12 reviews comprising 18,425 participants concluded a statistically significant reduction in body fat ranging from −1.6% to −2.6% (95% CI: −2.31 to −0.88) [73]. Yet, the other four systematic reviews concluded no difference between intervention and control groups. Therefore, longer interventions that include resistance exercise, behaviour change support (e.g., counselling) and other components (e.g., lifestyle advice, physical activity and dietary modification) might be more effective in changing body composition [73].

It should be considered that the results of the present study are likely to be influenced by differences in intervention adherence between groups. It is commonly reported that supervised exercise regimens typically elicit higher adherence than those that are unsupervised and remotely‐supported [74]. In the present study, adherence was higher in the partly‐supervised group (87% ± 7%) compared to the remotely‐supported group (64% ± 25%) which could have influenced the magnitude of change in the variables measured. Many predictors of adherence in exercise interventions have been identified which were not assessed in the present study, such as education, outcome expectations, motivations for exercise, lifetime exercise history and fatigue [75, 76]. Future research should address barriers to remotely‐supported exercise training regimens to design and implement interventions that have high adherence. It should also be considered that the present study did not include a non‐exercising control group, which might have provided a more robust analysis of intervention effects. However, a non‐exercising control group was not included for a variety of reasons (e.g., commonly reported control group improvement [77], ethical and recruitment considerations).

A strength of this study is the comparison of two matched exercise interventions that differed in their format of delivery and the type of support provided. Indeed, remotely‐supported exercise interventions might be more feasible than supervised interventions for adoption into mainstream cancer care. More broadly, remotely‐supported exercise is particularly relevant for larger scale public health interventions targeting for example, conditions such as overweight and obesity, type II diabetes and cardiovascular disease, given the ability to implement this format of intervention at scale and/or in underserved hard‐to‐reach communities. However, a weakness of the present remotely‐supported exercise intervention is the potential barrier that could come with a technology supported intervention (i.e., digital literacy) and the cost of appropriate devices and software. However, in the present study, and in the specific population of breast cancer survivors recruited from a hospital in the South‐West of England, features of the intervention, including use of so‐called smart watches, was supported by patient input during the design phase. Additional strengths of the present study include the combination of precise laboratory‐based measurements (e.g., directly measured cardiorespiratory fitness using indirect calorimetry, and detailed body composition assessment using DEXA) which were combined with simpler tests that could be used with minimal equipment and staff training in clinical settings (e.g., tests of physical functioning, such as the sit to stand test). It should also be considered that there may be outcomes beyond physical function measurements and body composition, that could be influenced by the delivery format of exercise interventions, including loneliness, isolation and social engagement. Indeed, a weakness of the present study is that broader elements of psychosocial wellbeing were not assessed, which could be important for appraising the utility of remotely‐supported compared to partly supervised interventions. For example, participants allocated to the remotely‐supported intervention in the present study did not have the twice‐weekly face‐to‐face social interaction that participants in the partly‐supervised group received. Thus, for some members of society, particularly those experiencing loneliness and social isolation, partly‐supervised interventions might be more beneficial and preferred.

## 5 Conclusions

In conclusion, the results of this study demonstrate that 8 weeks of remotely‐supported exercise among breast cancer survivors is an alternative to 8 weeks of partly‐supervised exercise specifically in relation to TUG test performance and aspects of body composition (BMI, percentage body fat, lean mass, BMD and BMD Z‐score and T‐score). In addition, the results of the study show that it remains inconclusive whether remotely‐supported exercise is an alternative to 8 weeks of partly‐supervised exercise when interpreting the effects of each intervention on cardiorespiratory fitness (V̇O_2_ max), blood pressure and other aspects of functional fitness (6MWT performance, sit to stand test score). Indeed, this study implies that remotely‐supported exercise interventions could be beneficial for breast cancer survivors. Barriers to unsupervised, remotely‐supported exercise interventions and practical applications for implementation require further investigation.

## Author Contributions

L.S.: conceptualisation, methodology, formal analysis, investigation, writing – original draft, visualisation, project administration. J.E.T.: conceptualisation,methodology, funding acquisition, supervision, writing – review and editing. D.T.: conceptualisation, methodology, funding acquisition, supervision, writing – review and editing. M.B.: supervision, writing – review and editing, investigation. J.B.: methodology, investigation, review and editing. J.P.W.: investigation.

## Funding

This work was supported by the University of Bath.

## Ethics Statement

Ethical approval was granted by an NHS research ethics committee (reference: 18/WA/0314). The study was performed in accordance with the ethical standards as laid down in the 1964 Declaration of Helsinki and its later amendments or comparable ethical standards.

## Consent

All participants provided written informed consent to participate.

## Conflicts of Interest

All authors declare that they have no competing interests. JAB is an investigator on research grants funded by BBSRC, MRC, British Heart Foundation, Rare Disease Foundation, EU Hydration Institute, GlaxoSmithKline, Nestlé, Lucozade Ribena Suntory, ARLA foods, Cosun Nutrition Center, American Academy of Sleep Medicine Foundation and Salus Optima (L3M Technologies Ltd); has completed paid consultancy for PepsiCo, Kellogg's, SVGC and Salus Optima (L3M Technologies Ltd); is Company Director of Metabolic Solutions Ltd.; receives an annual honorarium as a member of the academic advisory board for the International Olympic Committee Diploma in Sports Nutrition; and receives an annual stipend as Editor‐in Chief of *International Journal of Sport Nutrition & Exercise Metabolism*.

## Supporting information


**Data S1:** cam471608‐sup‐0001‐Supinfo1.docx.

## Data Availability

The data that support the findings of this study are available from the corresponding author upon reasonable request.
