# Supplementary material for: Cardiorespiratory Fitness, Functional Fitness and Body Composition Among Breast Cancer Survivors With 8 Weeks of Exercise Training: A Randomised, Controlled Non‐Inferiority Trial Comparing Remotely‐Supported and Partly‐Supervised Interventions
Source: Cancer Med. 2026 Feb 8;15(2):e71608. doi: 10.1002/cam4.71608 (PMC12883311; doi:10.1002/cam4.71608)
Supplement: Supplementary file 1 — Data S1: cam471608‐sup‐0001‐Supinfo1.docx. [file CAM4-15-e71608-s001.docx]

**Additional information**

**Additional Table 1.** Duration and intensity of exercise achieved

| **Exercise prescription** | | | | **Exercise achieved** | | | | | |
| --- | --- | --- | --- | --- | --- | --- | --- | --- | --- |
|  | | | | ***Partly-supervised*** | | | ***Remotely-supported*** | | |
| **Week** | **Duration** (minutes) | **Intensity**  (% V̇O_2_max) | **Intensity**  (approx. % HRmax) | **Total duration** (minutes) | **Intensity**  (HR, bpm) | **Intensity**  (approx. % HRmax) | **Total duration** (minutes) | **Intensity** (HR, bpm) | **Intensity**  (approx. % HRmax) |
| 1 | 105 | 55 | 71 | 104 ± 25 | 112 ± 16 | 69 ± 3 | 169 ± 103 | 114 ± 15 | 70 ± 11 |
| 2 | 105 | 55 | 71 | 110 ± 28 | 116 ± 18 | 72 ± 3 | 166 ± 106 | 113 ± 11 | 69 ± 7 |
| 3 | 120 | 60 | 74 | 111 ± 21 | 119 ± 13 | 73 ± 3 | 226 ± 129 | 114 ± 12 | 70 ± 8 |
| 4 | 120 | 60 | 74 | 133 ± 32 | 116 ± 14 | 72 ± 3 | 176 ± 116 | 112 ± 12 | 68 ± 10 |
| 5 | 135 | 65 | 78 | 142 ± 30 | 118 ± 10 | 73 ± 3 | 157 ± 102 | 115 ± 13 | 71 ± 9 |
| 6 | 135 | 65 | 78 | 135 ± 20 | 118 ± 10 | 73 ± 3 | 146 ± 96 | 115 ± 12 | 72 ± 6 |
| 7 | 150 | 70 | 81 | 141 ± 22 | 118 ± 8 | 73 ± 3 | 209 ± 94 | 119 ± 13 | 74 ± 6 |
| 8 | 150 | 70 | 81 | 138 ± 43 | 117 ± 13 | 72 ± 3 | 179 ± 128 | 119 ± 10 | 74 ± 6 |

*Table legend: Data shown as means ± SD. During supervised sessions in the partly-supervised group, the intensity of exercise (prescribed using VO2max thresholds) was checked and adjusted using indirect calorimetry. In the remotely-supported group and during the home-based exercise session in the partly-supervised group, the intensity of exercise was prescribed and monitored using heart rate targets corresponding to VO2max thresholds. For ease of interpretation, the achieved intensity is expressed as a percentage of age-predicted maximum heart rate.* V̇O_2_max*; maximum oxygen update HR; heart rate; bpm; beats per minute.*

**Additional Table 2.** Cardiorespiratory fitness and physical function pre- and post-exercise

|  |  | | **Pre** | **Post** | **Change**  (mean 95% CI) | **Main effect of time**  (one-way ANOVA) | **Interaction effect**  (two-way ANOVA) |
| --- | --- | --- | --- | --- | --- | --- | --- |
| **V̇O_2_max**  (ml.kg.min^-1^) | | Partly-supervised | 28.2 ± 3.9 | 28.7 ± 4.8 | 0.35 (−1.6 to 2.3) | F(1,14)=0.147, p=0.707 | F(1,28)=1.036, p=0.318 |
|  |  | Remotely-supported | 28.9 ± 6.8 | 28.2 ± 7.1 | −0.74 (−2.0 to 0.5) | F(1,14)=1.690, p=0.215 |  |
|  | |  |  |  |  |  |  |
| **Systolic blood pressure**  (mmHg) | | Partly-supervised | 135 ± 22 | 126 ± 22* | −9 (−14 to −4) | F(1,14)=12.965, p=0.003 | F(1,28)=1.341, p=0.257 |
|  |  | Remotely-supported | 125 ± 21 | 121 ± 19 | −5 (−11 to 2) | F(1,14)=2.382, p=0.145 |  |
|  | |  |  |  |  |  |  |
| **Diastolic blood pressure**  (mmHg) | | Partly-supervised | 80 ± 8 | 79 ± 10 | −2 (−6 to 3) | F(1,14)=0.841, p=0.375 | F(1,28)=0.042, p=0.840 |
|  |  | Remotely-supported | 16 ± 4 | 18 ± 5* | 2 (0 to 4) | F(1,14)=6.364, p=0.024 |  |
|  | |  |  |  |  |  |  |
| **6MWT**  (m) | | Partly-supervised | 475 ± 43 | 518 ± 35* | 43 (22 to 63) | F(1,14)=20.106, p=0.001 | F(1,28)=4.074, p=0.053 |
|  |  | Remotely-supported | 493 ± 72 | 509 ± 76 | 16 (−4 to 36) | F(1,14)=2.888, p=0.111 |  |
|  | |  |  |  |  |  |  |
| **Sit to stand**  (repetitions) | | Partly-supervised | 16 ± 4 | 19 ± 5* | 3 (1 to 5) | F(1,14)=7.166, p=0.018 | F(1,28)=0.322, p=0.575 |
|  |  | Remotely-supported | 16 ± 4 | 18 ± 5* | 2 (0 to 4) | F(1,14)=6.364, p=0.024 |  |
|  | |  |  |  |  |  |  |
| **TUG**  (seconds) | | Partly-supervised | 5.0 ± 0.8 | 4.8 ± 0.8 | −0.3 (−0.7 to 0.2) | F(1,14)=1.294, p=0.274 | F(1,28)=0.410, p=0.527 |
|  |  | Remotely-supported | 5.0 ± 1.1 | 5.0 ± 1.1 | −1 (−5 to 2) | F(1,14)=0.665, p=0.428 |  |

**Legend for additional table 2:** Data shown as means ± SD. * statistically significant difference from baseline, p<0.05. V̇O_2_max*; maximum oxygen update; 6MWT; 6 minute walk test, TUG; timed up and go test, kg; kilogram. min; minute. m; metres. mmHg; millimetres of mercury***.**

**Additional Table 3.** Body composition pre- and post-exercise.

|  |  | **Pre** | | **Post** | | **Change**  (mean (95% CI)) | | **Main effect of time**  (one-way ANOVA) | | **Interaction effect**  (two-way ANOVA) |
| --- | --- | --- | --- | --- | --- | --- | --- | --- | --- | --- |
| **BMI**  (kg.m^2^) | Partly-supervised | 25.8 ± 2.9 | | 25.9 ± 3.2 | | 0.1 (−0.4 to 0.6) | | F(1,14)=0.274, p=0.609 | | F(1,28)=0.086, p=0.772 |
|  | Remotely-supported | 24.4 ± 3.4 | | 24.4 ± 3.5 | | 0.0 (−0.3 to 0.4) | | F(1,14)=0.073, p=0.791 | |  |
|  |  |  | |  | |  | |  | |  |
| **Percentage body fat** (%) | Partly-supervised | 37.0 ± 3.9 | | 37.0 ± 3.6 | | −0.1 (−0.6 to 0.5) | | F(1,14)=0.171, p=0.685 | | F(1,28)=2.252, p=0.145 |
|  | Remotely-supported | 35.2 ± 7.3 | | 34.6 ± 7.6 | | −0.6 (−1.3 to 0.2) | | F(1,14)=2.827, p=0.115 | |  |
|  |  |  | |  | |  | |  | |  |
| **Lean mass**  (kg) | Partly-supervised | 41.7 ± 4.3 | | 41.9 ± 4.3 | | 0.2 (−0.4 to 0.8) | | F(1,14)=0.461, p=0.508 | | F(1,28)=0.205, p=0.654 |
|  | Remotely-supported | 42.2 ± 3.8 | | 41.6 ± 3.8 | | 0.3 (−0.3 to 0.8) | | F(1,14)=2.689, p=0.123 | |  |
|  |  |  | |  | |  | |  | |  |
| **BMD**  (g.cm^2^) | Partly-supervised | 1.128 ± 0.106 | | 1.112 ± 0.100 | | −0.016 (−0.040 to 0.008) | | F(1,14)=1.948, p=0.185 | | F(1,28)=1.054, p=0.313 |
|  | Remotely-supported | 1.098 ± 0.078 | | 1.096 ± 0.085 | | −0.009 (−0.027 to 0.011) | | F(1,14)=0.078, p=0.784 | |  |
|  |  |  | |  | |  | |  | |  |
| **Z-score** | Partly-supervised | 0.4 ± 1.1 | | 0.3 ± 0.9 | | −0.1 (−0.3 to 0.2) | | F(1,14)=0.215, p=0.650 | | F(1,28)=0.133, p=0.718 |
|  | Remotely-supported | 0.2 ± 0.7 | | 0.1 ± 0.7 | | −0.1 (−0.4 to 0.1) | | F(1,14)=0.133, p=0.718 | |  |
|  |  |  | |  | |  | |  | |  |
| **T-score** | Partly-supervised | 0.2 ± 1.3 | | 0.2 ± 1.2 | | −0.2 (−0.4 to 0.1) | | F(1,14)=1.592, p=0.650 | | F(1,28)=1.468, p=0.236 |
|  | Remotely-supported | −0.1 ± 1.0 | | −0.1 ± 0.9 | | −0.1 (−0.3 to 0.2) | | F(1,14)=0.093, p=0.765 | |  |
|  |  |  |  | |  | |  | |  | |

Legend for additional table 3: Data shown as means ± SD. * significant difference from baseline, p<0.05. *kg; kilogram. m; metres. g; grams. cm; centimetres. ANOVA; analysis of variance.*
